# Non-CG DNA Methylation Regulates Root Stem Cell Niche Maintenance, Auxin Signaling, and ROS Homeostasis in *Arabidopsis* Under Cadmium Stress

**DOI:** 10.3390/plants14182838

**Published:** 2025-09-11

**Authors:** Emanuela Talarico, Eleonora Greco, Fabrizio Araniti, Adriana Chiappetta, Leonardo Bruno

**Affiliations:** 1 Department of Biology, Ecology and Earth Sciences (DiBEST), Unit of Plant Biology, University of Calabria, Arcavacata of Rende, 87036 Cosenza, Italy; emanuela.talarico@unical.it (E.T.); eleonora.greco@unical.it (E.G.); adriana.chiappetta@unical.it (A.C.); 2 Department of Agricultural and Environmental Sciences-Production, Landscape, Agroenergy (Di.S.A.A.), University of Milano, 20133 Milan, Italy

**Keywords:** cadmium, DNA methylation, ROS, *Arabidopsis thaliana*, auxin, quiescent center

## Abstract

Non-CG DNA methylation plays a critical role in regulating root development and stress responses in *Arabidopsis thaliana* under cadmium (Cd^2+^) exposure. We compared wild type (WT) plants with the *ddc* triple mutant (deficient in *DRM1*, *DRM2*, and *CMT3*) to assess how epigenetic modifications affect the root apical meristem (RAM) under 100 µM and 150 µM CdCl_2_ treatments. Cd^2+^ exposure led to RAM disorganization, reduced cortical cell number, and quiescent center (QC) cell loss in WT roots, while *ddc* mutants maintained meristem integrity and exhibited QC cell expansion. Auxin signaling, assessed via *pDR5::GFP*, was disrupted in WT roots at high Cd^2+^ levels but remained stable in *ddc* mutants. Similarly, WT roots showed elevated reactive oxygen species accumulation under stress, whereas *ddc* mutants displayed a reduced oxidative response. These results suggest that non-CG DNA methylation suppresses key regulators of stem cell maintenance, hormonal balance, and redox homeostasis during heavy metal stress. Loss of this methylation in the *ddc* mutant confers enhanced resilience to Cd^2+^ toxicity, highlighting an epigenetic mechanism underlying root stress adaptation.

## 1. Introduction

Cadmium (Cd^2+^) is a non-essential, highly toxic heavy metal with no known biological function in plants. It represents a major environmental pollutant, largely originating from anthropogenic activities such as mining, metal smelting, industrial emissions, and excessive use of phosphate fertilizers [1]. Once present in the soil, Cd^2+^ can be readily taken up by plant roots due to the relatively high mobility and solubility of certain cadmium compounds (e.g., cadmium chloride, nitrate, and sulfate), leading to its accumulation in vegetative tissues and, ultimately, entry into the food chain [2]. However, it is important to note that other cadmium salts, such as cadmium sulfide and cadmium oxide, are sparingly soluble or insoluble, which can limit their bioavailability.

Cd^2+^ exposure interferes with multiple physiological and biochemical processes in plants, including nutrient uptake, photosynthesis, respiration, and water relations, ultimately causing stunted growth, chlorosis, necrosis, and reduced productivity [3,4,5].

Among all plant organs, roots are the first and most directly affected by Cd^2+^ due to their contact with the contaminated rhizosphere. In *Arabidopsis thaliana* (L.) *Heynh,* Cd^2+^ concentrations in the range of 100 to 150 µM significantly inhibit primary root elongation, impair lateral root formation, and disrupt root and shoot apical meristem organization [6,7]. These morphological defects are closely associated with changes in cell division and differentiation, cytoskeletal organization, and stem cell niche integrity. The root tip and elongation zones, being sites of intense cell proliferation and elongation, are particularly sensitive to external perturbations and are regulated by hormonal gradients, primarily auxin and cytokinin [8]. Similarly, short-term metabolic reprogramming in maize roots has been observed upon exposure to trans-cinnamic acid, highlighting the sensitivity of root metabolism to external chemical cues [9].

Cd^2+^ stress rapidly triggers the accumulation of reactive oxygen species (ROS), notably superoxide radicals (O_2_•^−^) and hydrogen peroxide (H_2_O_2_), which are among the earliest responses detectable within seconds of exposure [10,11]. Though Cd^2+^ is not redox-active under physiological conditions and does not participate directly in Fenton-type reactions like copper (Cu) or iron (Fe), it indirectly promotes oxidative stress by interfering with antioxidant systems and enhancing ROS production [12]. ROS can damage proteins, lipids, and nucleic acids, but they also function as signaling molecules modulating stress responses. Their spatial and temporal distribution, particularly in root tissues, is tightly regulated [13].

Importantly, ROS are intricately linked to hormonal pathways, especially auxin. Cd^2+^ disrupts auxin biosynthesis, transport, and signaling, notably by impairing the polar localization and expression of PIN-FORMED (PIN) auxin efflux carriers. This leads to disturbed auxin gradients and abnormal root patterning [7,14], which is also affected by other natural compounds, such as coumarin [15]. Auxin also regulates ROS scavenging enzymes and NADPH oxidase activity, forming a bidirectional feedback loop between ROS and hormone signaling [16]. The interaction between ROS and auxin under Cd^2+^ stress thus adds a layer of complexity to the regulation of root development and stress responses.

In recent years, increasing attention has been devoted to the role of epigenetic mechanisms, especially DNA methylation, in modulating plant responses to abiotic stresses, including heavy metal toxicity. DNA methylation involves the addition of a methyl group to cytosine residues in CG, CHG, and CHH sequence contexts and is mediated by distinct classes of enzymes. CG methylation, primarily maintained by METHYLTRANSFERASE 1 (MET1), ensures the faithful inheritance of epigenetic information during DNA replication. In contrast, non-CG methylation at CHG and CHH sites is maintained by CHROMOMETHYLASEs (CMTs)—which contain a CHROMO (CHRomatin Organization Modifier) domain that recognizes methylated histones and links DNA methylation to chromatin structure—and is established de novo by DOMAINS REARRANGED METHYLTRANSFERASEs (DRMs). Together, these pathways play a central role in silencing transposable elements and regulating gene expression [17]. Environmental stresses induce dynamic changes in DNA methylation patterns, leading to transcriptional reprogramming [18]. For example, it has been observed that DNA methylation positively regulate the growth of hypocotyl under heat stress by influencing auxin levels and GA biosynthesis [19]. The *Arabidopsis ddc* mutant, which lacks two key DNA methyltransferases and one chromomethylase (*DRM1*, *DRM2*, and *CMT3*), displays severe defects in non-CG methylation and altered responses to environmental stressors [20]. It is characterized by reduced stature, developmental delay, curly leaves, and partial sterility—unlike single *drm1 drm2* or *cmt3* mutants, which are phenotypically normal. Auxin accumulation and distribution across embryo, leaf, and root tissues are altered. Transcriptomic profiling indicates misexpression of genes involved in auxin biosynthesis, transport, and signaling, demonstrating a direct link between non-CG methylation and auxin pathways [20,21]. Under Cd^2+^ stress, *ddc* displays enhanced tolerance compared to wild type (WT), maintaining longer roots and larger rosettes [22]. Notably, Pacenza et al. [22] demonstrated that, in response to Cd^2+^ stress, the *ddc* mutant exhibits an adaptive strategy oriented towards the maintenance of growth hormones rather than the prolonged activation of stress-response hormones, suggesting a relevant role of DNA methylation in the epigenetic regulation of hormone balance and plant survival. ROS can also influence the activity of DNA methyltransferases and demethylases, providing a potential feedback loop between oxidative stress and epigenetic regulation [23]. Therefore, ROS, auxin, and DNA methylation represent a tightly interconnected network of signaling and regulatory components activated under Cd^2+^ stress. To better understand Cd^2+^’s effects on root development, researchers have used model species like *Arabidopsis thaliana* and *Oryza sativa*, including transgenic lines [6,24]. Bruno et al. [7] demonstrated that short-term exposure to high Cd^2+^ concentrations disrupts shoot and root meristems by altering the expression of *WUSCHEL (WUS)/WOX* genes and causing cytokinin accumulation. Additional studies have shown that Cd^2+^ affects root radial patterning by misregulating the *SCARECROW* (SCR) transcription factor and disturbing the auxin–cytokinin balance, thereby inhibiting primary root elongation even at relatively low concentrations [6].

Cd^2+^ is a strong stress factor that also affects the development of the rice (*Oryza sativa*) root system, mainly by interfering with auxin metabolism and distribution. Ronzan et al. [25] show that Cd^2+^ reduces endogenous IAA levels and alters the expression of key auxin homeostasis genes, causing a halt in lateral and adventitious root formation.

In this study, we investigated the effects of acute Cd^2+^ stress on *Arabidopsis thaliana* roots by applying high Cd^2+^ concentrations (100 and 150 µM) for a short duration (24 h). We focused on changes in root morphology, auxin distribution, and reactive oxygen species (ROS) accumulation to gain insight into the spatial dynamics of oxidative stress under Cd^2+^ exposure. By comparing WT and *ddc* mutant plants, we aimed to elucidate the role of DNA methylation in modulating oxidative and hormonal signaling pathways in the root system. Our results reveal that *ddc* mutants exhibit altered ROS accumulation patterns compared to WT, potentially due to epigenetically driven misregulation of genes involved in ROS perception, detoxification, and hormone signaling. These findings deepen our understanding of the epigenetic regulation of root stress responses and may inform strategies for enhancing crop resilience to heavy metal contamination.

## 2. Results

### 2.1. Cadmium Exposure Inhibits Primary Root Growth in Both WT and ddc Seedlings

**Figure 1:**
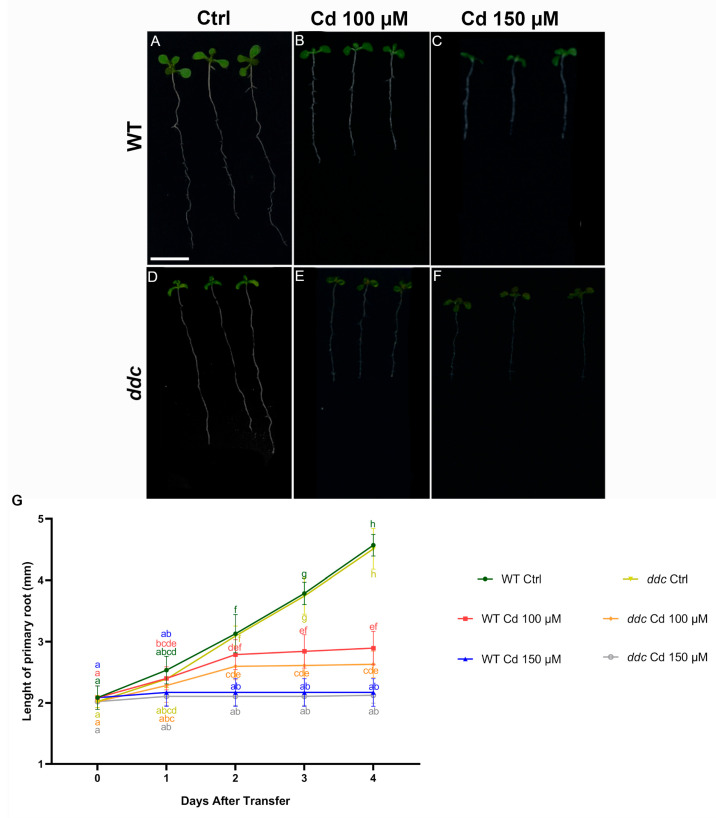
(**A**–**F**) Whole-root morphology of *Arabidopsis thaliana* wild type (**A**–**C**) and *ddc* mutant (**D**–**F**) seedlings. Seedlings were grown for 5 days on control medium and then transferred for 24 h to (**A**,**D**) control medium, (**B**,**E**) 100 μM Cd^2+^, or (**C**,**F**) 150 μM Cd^2+^. (**G**) Primary root length. Data represent the mean ± standard deviation of three independent experiments. Statistical analysis was performed using ANOVA followed by Tukey’s test (*p* < 0.05); samples sharing the same letter are not significantly different. (**A**–**F**) Scale bars: 1 cm. N = 50.

To investigate the effects of Cd^2+^ toxicity on root development, WT and *ddc* mutant seedlings were transferred to control medium or medium supplemented with 100 μM or 150 μM Cd^2+^. After four days of treatment, root growth was markedly impaired in both genotypes compared with their respective controls (Figure 1A–F).

Under control conditions, WT seedlings developed long primary roots, while *ddc* mutants displayed slightly shorter roots, although the difference was not statistically significant at early time points (Figure 1G). Treatment with 100 μM Cd^2+^ significantly reduced primary root elongation in both WT and *ddc* compared to their untreated controls. The inhibitory effect was even more pronounced at 150 μM Cd^2+^, where primary root growth was nearly arrested (Figure 1B,C,E,F).

Quantitative analysis of root length confirmed these observations (Figure 1G). WT seedlings under Ctrl conditions showed a progressive and significant increase in root length over time, reaching ~4.5 mm by day 4. In contrast, Cd-treated WT seedlings exhibited strongly reduced root elongation, with roots reaching only ~2.9 mm and ~2.2 mm at 100 μM and 150 μM Cd, respectively. Similarly, *ddc* mutants exhibited inhibited root growth under Cd exposure. While *ddc* control seedlings grew comparably to WT controls, their response to Cd stress showed a consistent reduction in elongation, with roots under 100 μM Cd reaching ~2.6 mm and those under 150 μM Cd remaining around ~2.1 mm after 4 days.

Statistical analysis revealed significant differences among treatments and genotypes across time points (Figure 1G). Notably, while both genotypes were sensitive to Cd stress, *ddc* mutants did not display enhanced tolerance compared to WT, suggesting that the *ddc* mutation does not confer protection against Cd-induced root growth inhibition.

### 2.2. Non-CG DNA Methylation Affects Quiescent Center Dynamics Under Cadmium Stress

**Figure 2:**
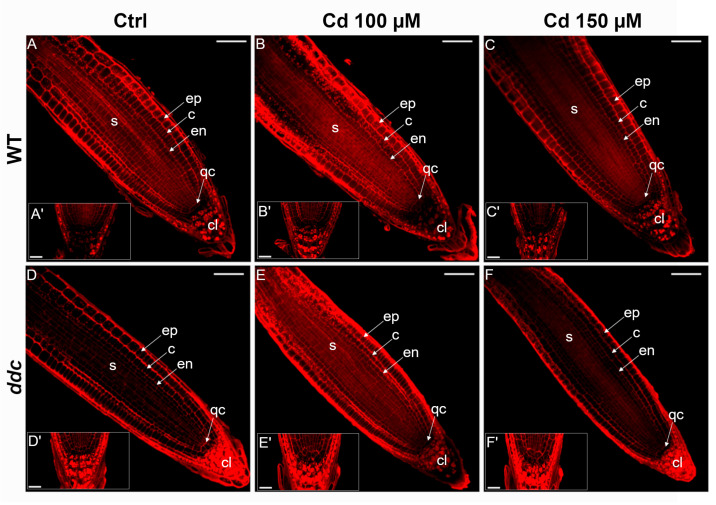
Confocal laser images of the primary root meristem (**A**–**F**) and close-ups of the quiescent center (**A′**–**F′**) in *Arabidopsis thaliana* wild type (**A**–**C**) and *ddc* mutant (**D**–**F**) seedlings grown for 5 days on control medium and then transferred for 24 h to (**A**,**D**) control medium, (**B**,**E**) 100 μM Cd^2+^, or (**C**,**F**) 150 μM Cd^2+^. c, cortex; cl, columella; en, endodermis; ep, epidermis; qc, quiescent center; s, stele. (**A**–**F**) Scale bar: 50 μm; (**A′**–**F′**): 20 μm. N = 50.

**Figure 3:**
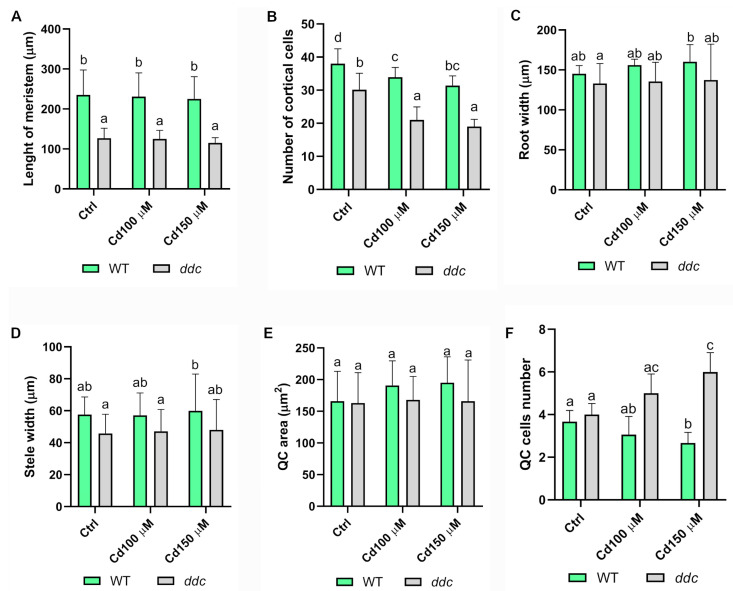
Morphometric analysis of Root Apical Meristem (RAM) in *Arabidopsis thaliana* seedlings (WT and *ddc*) grown for 5 days on control medium and then transferred for 24 h to control medium, 100 μM Cd^2+^, or 150 μM Cd^2+^. (**A**) Meristem length (μm), (**B**) cortical cells number, (**C**,**D**) root and stele width (μm), (**E**) area of quiescent center (μm^2^), (**F**) number of cells in quiescent center in both genotypes. Data present the mean ± standard deviation of three independent experiments. Values were analyzed with ANOVA and Tukey’s rank test (*p* < 0.05). Samples marked with the same letter do not present significant differences. N = 50.

To investigate the role of non-CG DNA methylation in modulating root architecture under Cd^2+^ stress, we analyzed the organization of the RAM in *Arabidopsis thaliana* WT and *ddc* triple mutant plants (Figure 2 and Figure 3). Confocal laser scanning microscopy was performed on root tips stained with propidium iodide under control conditions and following 24 h exposure to 100 or 150 µM Cd^2+^ (Figure 2A–F).

Meristem length (Figure 3A) remained largely unchanged across treatments in both genotypes. However, a significant reduction in the number of cortical cell files was observed in both WT and *ddc* under Cd^2+^ stress (Figure 3B). Notably, root (Figure 3C) and stele width (Figure 3D) were unaffected by Cd^2+^ treatment in either genotype, indicating that radial patterning is maintained even though longitudinal growth is altered. In this context, longitudinal growth is largely driven by cortical cell files, and a reduction in the number of cells within these files is directly associated with decreased root elongation.

Closer examination of the quiescent center (QC) revealed genotype-specific responses (Figure 2A′–F′). While QC area (Figure 3E) remained stable, WT roots exhibited a significant reduction in QC cell number at 150 µM Cd^2+^ (Figure 3F), suggesting a potential loss of QC identity. In contrast, *ddc* mutants showed a significant increase in QC cell number at both Cd^2+^ concentrations (Figure 3F), implying expansion or stabilization of the QC population in the absence of non-CG methylation.

These findings support a model in which non-CG DNA methylation contributes to the repression of stem cell regulatory networks under heavy metal stress. Loss of this epigenetic regulation in the *ddc* mutant appears to preserve RAM organization and QC integrity, potentially enhancing root resilience under Cd^2+^ exposure.

### 2.3. Differential Auxin Signaling Response to Cadmium Stress in Wild Type and ddc Mutant Roots

**Figure 4:**
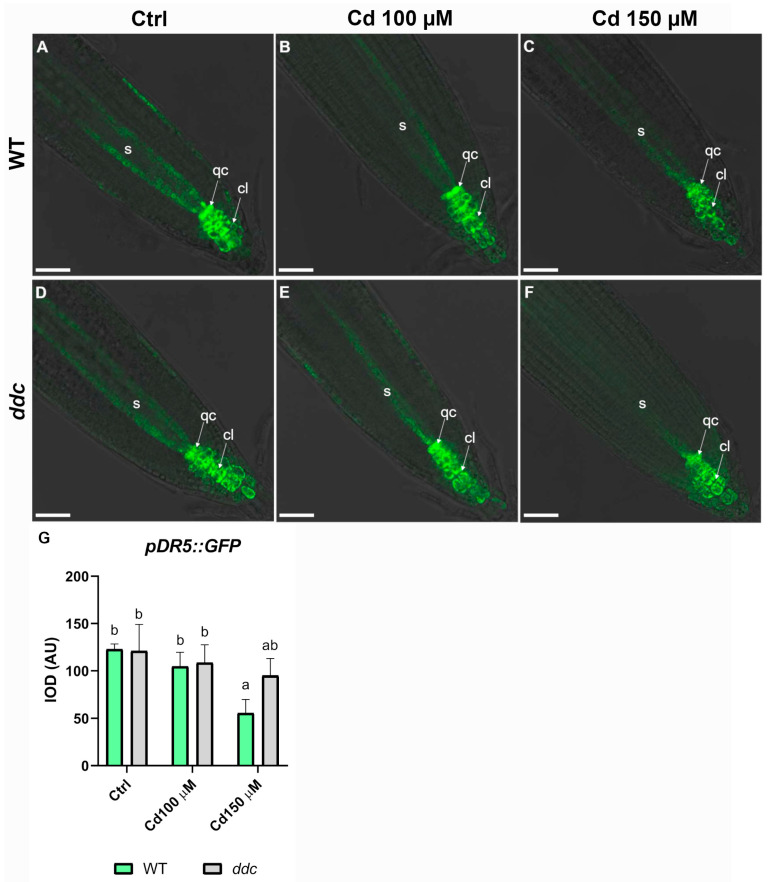
Confocal laser images of the apical root meristem in *Arabidopsis thaliana pDR5::GFP* transgenic lines in wild type (**A**–**C**) and *ddc* (**D**–**F**) backgrounds, grown for 5 days on control medium and then transferred for 24 h to (**A**,**D**) control medium, (**B**,**E**) 100 μM Cd^2+^, or (**C**,**F**) 150 μM Cd^2+^. (**G**) Integrated optical density (IOD) expressed as arbitrary units (AU) of fluorescence intensity in wild type and *ddc* mutant. Data present the mean ± standard deviation of three independent experiments. Values were analyzed with ANOVA and Tukey’s rank test (*p* < 0.05). Samples marked with the same letter do not present significant differences. cl, columella; qc, quiescent center; s, stele. Scale bar: 50 μm. N = 50.

Given the previously observed genotype-specific alterations in the QC under Cd^2+^ stress and the well-established role of auxin in maintaining QC identity and function, we analyzed the expression of the auxin-responsive *pDR5::GFP* reporter in the RAM of *Arabidopsis thaliana* WT and *ddc* mutant plants exposed to increasing Cd^2+^ concentrations (100 µM and 150 µM) (Figure 4). In WT roots under Ctrl conditions, GFP signal was strongly localized to the QC and surrounding columella cells, reflecting the characteristic auxin maximum at the root tip (Figure 4A). This pattern was largely maintained under 100 µM Cd^2+^ treatment (Figure 4B); however, exposure to 150 µM Cd^2+^ (Figure 4C) led to a marked reduction in GFP intensity, suggesting a disruption of auxin accumulation or signaling in the root apex. In contrast, *ddc* mutant roots displayed a comparable *pDR5::GFP* pattern under all treatment conditions. Both Ctrl (Figure 4D) and Cd^2+^-treated roots (100 µM and 150 µM; Figure 4E,F) maintained strong GFP fluorescence in the root tip, indicating that auxin maxima were preserved even under higher Cd^2+^ stress. Quantification of GFP signal intensity (IOD, Integrated Optical Density) confirmed these observations. In WT, a statistically significant reduction in *pDR5::GFP* intensity was observed at 150 µM Cd^2+^ compared to Ctrl and 100 µM Cd^2+^ treatments (Figure 4G). In contrast, *ddc* roots showed no significant differences in *pDR5::GFP* intensity across treatments (Figure 4G), suggesting that auxin signaling is less affected by Cd^2+^ exposure in the *ddc* mutant. These findings indicate that the *ddc* mutant exhibits enhanced stability of auxin signaling under Cd^2+^ stress, potentially due to altered epigenetic regulation. The preservation of *pDR5::GFP* expression in *ddc* roots suggests a protective role of disrupted non-CG DNA methylation in maintaining auxin-dependent root meristem activity under metal toxicity.

### 2.4. ROS Differentially Accumulate in Wild Type and ddc Mutant Roots Under Cadmium Stress

**Figure 5:**
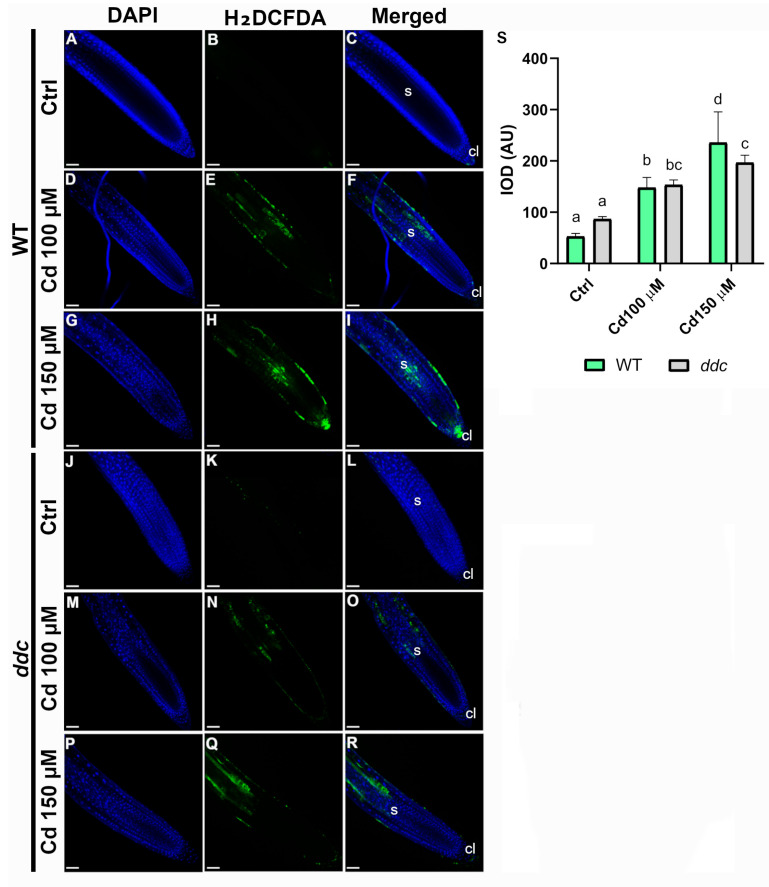
ROS staining in the primary root of *Arabidopsis thaliana* wild type (**A**–**I**) and *ddc* mutant (**J**–**R**). Seedlings were grown for 5 days on control medium and then transferred for 24 h to (**A**–**C**,**J**–**L**) control medium, (**D**–**F**,**M**–**O**) 100 μM Cd^2+^, or (**G**–**I**,**P**–**R**) 150 μM Cd^2+^. ROS (green color) and nuclei counterstained with DAPI (blue color) were visualized using confocal microscopy. (**A**,**D**,**G**,**J**,**M**,**P**) ROS channel; (**B**,**E**,**H**,**K**,**N**,**Q**) DAPI channel; (**C**,**F**,**I**,**L**,**O**,**R**) merged images. (**S**) Integrated optical density (IOD), expressed as arbitrary units (AU), of ROS fluorescence in wild type and *ddc*. Data present the mean ± standard deviation of three independent experiments. Values were analyzed with ANOVA and Tukey’s rank test (*p* < 0.05). Samples marked with the same letter do not present significant differences.cl, columella; s, stele. Scale bar: 50 μm. N = 50.

To evaluate the oxidative response to Cd^2+^ exposure, ROS accumulation was assessed in *Arabidopsis thaliana* WT and *ddc* root tips treated with 100 µM and 150 µM Cd^2+^ (Figure 5). In WT roots, no ROS signal was detected under Ctrl conditions (Figure 5A–C). At 100 µM Cd^2+^, moderate green fluorescence was detected, with signal localized primarily along the root epidermis and in discrete internal cell layers, particularly in the meristematic and elongation zones (Figure 5D–F). The distribution suggests a localized oxidative response, likely associated with early stress signaling. In contrast, treatment with 150 µM Cd^2+^ led to a marked increase in ROS signal intensity and distribution. Strong fluorescence was observed in the root cap, cortical tissues, and epidermis, extending into the elongation zone (Figure 5G–I), indicating a more pronounced oxidative stress, possibly impairing root growth and development. In *ddc* roots, Ctrl conditions showed no detectable ROS signal (Figure 5J–L), similar to WT. However, at 100 µM Cd^2+^, it displayed low ROS signal, with only faint fluorescence detected in the root apex and elongation zone (Figure 5M–O). Next, 150 µM Cd^2+^ treatment triggered an increase in ROS accumulation in the *ddc* roots (Figure 5P–R), although weaker compared to WT, as confirmed by the quantification of fluorescence signal (Figure 5S). The fluorescence signal was evident particularly in the root cap and columella, as well as in the cortical cell layers near the apex. Overall, these findings demonstrated that Cd^2+^ induces ROS accumulation in a concentration-dependent manner, stronger in WT compared to *ddc* mutant, suggesting a muted oxidative response at these Cd^2+^ concentrations and thus a possible role for DNA methylation in modulating ROS-mediated stress signaling and root sensitivity to Cd^2+^.

## 3. Discussion

Our results reveal a pivotal role for non-CG DNA methylation in regulating root meristem structure, hormonal signaling, and oxidative stress responses in *Arabidopsis thaliana* under Cd^2+^ stress. By comparing WT plants with the *ddc* triple mutant deficient in the DNA methyltransferases *DRM1*, *DRM2*, and *CMT3* responsible for non-CG methylation we demonstrate that the plant’s epigenetic landscape profoundly influences its capacity to maintain RAM integrity, preserve hormonal homeostasis, and mitigate oxidative damage under toxic heavy metal exposure.

Our growth kinetics data show that primary root elongation in both WT and *ddc* seedlings is strongly inhibited under Cd^2+^ exposition. Specifically, the *ddc* mutant displays a shorter root length compared to WT, even under Ctrl conditions, which is consistent with its baseline phenotype. In addition, the Cd^2+^-induced reduction in meristem length and cortical cell number is comparable between the two genotypes, suggesting that the *ddc* mutation does not confer a significant advantage in terms of preservation of global root cyto-histological architecture. It is important to note, however, that this short-term, high-concentration Cd^2+^ treatment represents an acute stress scenario associated with oxidative challenges and may not fully reflect the effects of chronic, environmentally relevant Cd^2+^ exposure. Our observations complement prior work by Pacenza et al. [22], who reported Cd^2+^-induced root elongation inhibition in both WT and *ddc* backgrounds, highlighting the physiological significance of RAM maintenance in modulating stress responses.

However, examination of the QC, a critical domain for root stem cell maintenance [26], revealed distinct genotype-specific responses. Although the QC area remained stable regardless of treatment, WT plants experienced a significant decrease in QC cell number under Cd^2+^ stress, implying a loss of stem cell identity or viability. Remarkably, *ddc* mutants showed an increase in QC cell numbers at both 100 µM and 150 µM Cd^2+^, indicating an increase in mitotic activity, even in cells known to be inactive [27], under Cd^2+^-stress in a hypomethylated background. This divergent behavior likely reflects epigenetic regulation of stem cell niche dynamics, where probably non-CG methylation acts as a repressive mechanism on genes essential for QC maintenance and stemness. The sensitivity of the QC to Cd^2+^ toxicity has been documented previously. Bruno et al. [6] demonstrated that Cd^2+^ disrupts stem cell identity by altering auxin gradients within the RAM, leading to QC cell death and impaired meristem function. Similarly, Fattorini et al. [28] reported that Cd^2+^ exposure triggers hormonal imbalance, causing disorganization and loss of QC cells in *Arabidopsis* roots. These studies support our observations and highlight the crucial interplay between heavy metal stress, epigenetic regulation, and stem cell niche maintenance. Our findings align with additional literature showing that DNA methylation changes enable plants to modulate hormonal signaling pathways and oxidative stress responses under abiotic challenges [18]. The protective effects observed in *ddc* mutants underscore the importance of epigenetic plasticity in sustaining root development and stress tolerance mechanisms, offering novel insights into plant adaptation strategies against heavy metal toxicity. In WT roots, exposure to high Cd^2+^ concentrations (150 µM) disrupted the characteristic auxin maxima in the QC and columella, a pattern consistent with Cd^2+^-induced interference in auxin biosynthesis, polar transport, and PIN protein localization, as reported in earlier studies [6,7,14]. This disruption likely impairs the proper functioning of the root apical meristem (RAM), affecting root patterning and growth. In contrast, the *ddc* mutant preserved a robust *pDR5::GFP* signal across both Cd^2+^ treatments, suggesting that auxin distribution and/or responsiveness is more resilient in the absence of DRM and CMT3 mediated DNA methylation. The preservation of DR5 expression in the *ddc* mutant under Cd^2+^ stress not only highlights auxin signaling stability but may also underlie the maintenance of meristematic identity and activity [29]. In *Arabidopsis*, the QC and surrounding stem cell niche are maintained through auxin-dependent transcriptional programs involving *WUSCHEL-related homeobox 5* (*WOX5)*, *PLETHORA* (*PLT*), and *SCARECROW* (*SCR*) genes [30,31,32,33]. It is plausible that the ability of *ddc* to sustain a strong auxin maximum under Cd^2+^ exposure contributes to the preservation of root apical meristem architecture and cellular organization. Consistent with this knowledge, histological analyses revealed a genotype-specific trend in the QC area under Cd^2+^ stress; while WT type roots exhibited a marked reduction in QC number, *ddc* mutants showed a tendency toward increased. Given the central role of the QC in maintaining the surrounding stem cell niche, this observation suggests that the hypomethylated epigenetic background of *ddc* may directly promote the expansion or stabilization of QC cell identity under stress conditions. Notably, *WOX5* expression is required for maintaining QC identity and repressing differentiation of distal stem cells, and its promoter activity has been shown to be modulated by epigenetic regulation which determine chromatin accessibility [34]. The observed increase in QC cell number in *ddc* may thus reflect the relief of non-CG methylation-mediated repression at these loci, enabling continued expression of developmental regulators that preserve RAM organization and functionality under Cd^2+^-induced stress. In this context, non-CG DNA methylation might act as a repressive layer over key meristem maintenance genes under stress conditions, and its absence in *ddc* may allow continued expression of these developmental regulators. Notably, the radial organization of the root, including the width of the transition zone and the number of cell files in the stele, remained unaffected by Cd^2+^ treatments in both lines. This preservation of radial patterning suggests that Cd^2+^-induced perturbations primarily affect longitudinal growth and meristem activity, while the fundamental tissue architecture is maintained, possibly as a protective strategy. These results suggest that, despite preserved auxin signaling, the root architecture in *ddc* is still responsive to Cd^2+^ toxicity, though in a quantitatively distinct manner compared to WT.

Furthermore, the preserved auxin maxima observed in *ddc* roots was accompanied by lower ROS accumulation, which may contribute to maintaining QC identity and preventing cell death [35], in contrast to the WT where Cd^2+^ induced a partial loss of the QC. These results support a role for DNA methylation in stabilizing the stem cell niche by repressing unwanted proliferation and enabling stress-buffering mechanisms. Indeed, in WT roots, we observed a dose-dependent increase in ROS signal, with a broad and intense distribution at 150 µM Cd^2+^, particularly in the root cap and elongation zone. This is consistent with Cd^2+^-induced oxidative stress and the generation of ROS such as hydrogen peroxide and O_2_•^−^, which can cause severe cellular damage if not properly controlled [13]. In *ddc* roots, ROS levels were significantly lower at 100 µM Cd^2+^ and showed a more confined spatial distribution even at 150 µM, suggesting that loss of DNA methylation influences the plant’s redox homeostasis. The attenuated ROS response in *ddc* could result from multiple, non-mutually exclusive mechanisms, including downregulation of ROS-generating enzymes, upregulation of antioxidant systems, or altered hormonal feedback that modulates redox signaling, especially given the strong auxin ROS crosstalk. Cd^2+^-induced ROS production is known to involve both enhanced generation via NADPH oxidases such as RBOHD and impaired detoxification, due to inhibition or downregulation of antioxidant enzymes including CATALASE, ASCORBATE PEROXIDASE, and GLUTATHIONE PEROXIDASE [36,37,38,39,40]. It is therefore plausible that the attenuated ROS response in *ddc* reflects either enhanced expression of ROS scavenging enzymes or reduced activation of ROS producing systems, due to the loss of repressive non-CG methylation marks. This could explain why *ddc* mutants maintain lower ROS levels at moderate Cd^2+^ concentrations, potentially conferring a protective advantage at the cellular level. Auxin itself can influence ROS-scavenging gene expression, while ROS regulate auxin transport and biosynthesis, forming a complex bidirectional loop [41]. Moreover, these findings align with the model proposed by Pacenza et al. [22], where *ddc* mutants, under Cd^2+^ stress, prioritize the maintenance of growth-promoting hormones such as auxins, cytokinins, and gibberellins over stress-associated hormones like abscisic acid, jasmonates, and salicylic acid. This hormonal strategy may help to minimize the detrimental effects of prolonged defense activation, supporting continued development under adverse conditions. Importantly, the epigenetic regulation of this hormonal switch appears central to stress adaptability and could function as a molecular decision-making node between growth and defense, the so-called “life-or-death switch” in plants. Together, our data support a model in which non-CG methylation contributes to environmental sensitivity by controlling transcriptional programs that govern hormone signaling and oxidative stress. The *ddc* mutant, lacking this epigenetic repression, displays enhanced tolerance likely due to the sustained expression of auxin-responsive and stress-buffering genes [42]. This reinforces the emerging view that epigenetic flexibility enhances phenotypic plasticity, enabling plants to better adjust to rapid environmental changes. Importantly, our work contributes to the growing body of evidence that DNA methylation is not merely a static silencing mechanism but a dynamic and reversible regulator of gene expression in response to stress cues. Under heavy metal exposure, epigenetic modifications such as methylation may act as critical integrators of developmental and defense pathways, offering a means to reprogram plant responses in real-time. The application of this knowledge could inform breeding strategies or biotechnological interventions aimed at enhancing stress tolerance in crops cultivated in contaminated or degraded soils.

## 4. Materials and Methods

### 4.1. Plant Materials and Growth Conditions

Seeds of *Arabidopsis thaliana* WT ecotype Columbia-0 (Col-0), *ddc* triple mutant (defective in *DRM1 DRM2* and *CMT3*) and *pDR5::GFP* transgenic line of both genotypes (a synthetic auxin-inducible promoter fused to the GFP reporter) were surface-sterilized following the protocol described in Araniti et al. [14].

The *pDR5::GFP* seeds in the *ddc* background were obtained by crossing *ddc* mutants with the *pDR5::GFP* Col-0 line, as described in Forgione et al. [20].

The identity of the *ddc* triple mutant and the presence of the *pDR5::GFP* transgene in this background were previously confirmed by PCR genotyping and GFP fluorescence analysis, as described in Forgione et al. [20].

Seeds were surface-sterilized, sown on half-strength Murashige and Skoog (½ MS) agar plates, and stratified at 4 °C in the dark for 3 days to synchronize germination. Then, plates were moved in under long-day conditions (16 h light/8 h dark) at 22 °C and positioned vertically. After germination, seedlings were maintained for five days on a standard agar-based control medium (Ctrl). Subsequently, they were transferred to media supplemented with cadmium chloride (CdCl_2_) at final concentrations of 100 µM or 150 µM. Ctrl roots were maintained on Cd^2+^-free medium. Each treatment was replicated three times independently, with at least 50 seedlings analyzed per replicate.

### 4.2. Analysis of Root Growth Parameters

Primary root length was monitored daily in plants grown for 5 days in Ctrl conditions and then transferred to media containing 100 or 150 µM CdCl_2_, from the day of transfer (0 Days After Transfer, DAT) to day 4. Measurements were performed using image analysis with ImageJ software (https://imagej.net/ij/, accessed on 5 April 2025) by scanning the plates. Additionally, at 24 h post-transfer, the following parameters were evaluated: meristem length, number of cortical cells, root and stele width, QC area, and number of QC cells, all quantified through ImageJ-based image analysis.

### 4.3. Histochemical Staining (mPS-PI Staining)

To consider the architecture of meristematic cells in primary root, *Arabidopsis* seedlings (Col-0 and *ddc* mutant) grown on Ctrl medium and exposed for 24 h to Cd^2+^ (100 and 150 µM) 5 days after germination were used for mPS-PI Staining as described in Truernit et al. [43]. Briefly, seedlings were fixed in 50% methanol and 10% acetic acid at 4 °C overnight and then were transferred to 80% ethanol and incubated at 80 °C for 5 min. Successively, the seedlings were transferred back to the fixative for 60 min. After washing steps in sterile distilled water, samples were transferred into 1% periodic acid at room temperature for 40 min and then washed again with water. Seedlings were incubated in Schiff reagent (100 mM sodium metabisulphite and 0.15 N HCl; propidium iodide to a final concentration of 100 mg/mL was added at the moment) for 2 h and then were transferred onto microscope slides and covered with a chloral hydrate solution. Three independent replicates were performed, and a minimum of 40 seedlings was analyzed for each sample.

### 4.4. Confocal Microscopy Analysis of GFP Signal Localization

The localization and intensity of GFP signal were examined in the synthetic auxin response reporter DR5 (*pDR5::GFP*). Seedlings were grown and treated as described above. Imaging was performed using a Leica TCS SP8 inverted confocal laser scanning microscope equipped with a 40× oil immersion objective. GFP excitation was achieved with a 488 nm argon laser, and emission was detected at 509 nm, as described in Bruno et al. [6]. Fluorescence intensity was quantified using ImageJ software (https://imagej.net/ij/). All experiments were conducted in triplicate, with a minimum of 50 seedlings analyzed per condition.

### 4.5. Reactive Oxygen Species Detection

Intracellular accumulation of ROS in root tissues was visualized using a fluorescent ROS-sensitive dye. Five-day-old *Arabidopsis* seedlings (both Col-0 and *ddc*) were grown and treated as described above. After treatment, roots were incubated with 25 µM H_2_DCFDA (2′,7′-dichlorodihydrofluorescein diacetate; Image-iT^TM^ LIVE Green ROS Detection Kit, Invitrogen™ Molecular Probes, Thermo Fisher Scientific, Waltham, MA, USA), following manufacturer’s instructions. Staining was performed in the dark for 20 min. at 37 °C. Following incubation, seedlings were rinsed three times in PBS to remove excess dye and immediately mounted on microscope slides in PBS for imaging. Other nuclei were counterstained with DAPI (4′,6-Diamidino-2 Phenylindole, Dihydrochloride, Invitrogen™ Molecular Probes, Thermo Fisher Scientific, Waltham, MA, USA). Confocal images were acquired using a Leica TCS SP8 inverted confocal laser scanning microscope with a 20× objective. The ROS dye was excited at 488 nm with an argon laser, and the emitted fluorescence was collected between 500 and 530 nm, whereas the DAPI at 358 nm and at 461 nm respectively. All imaging parameters (gain, laser intensity, exposure time) were kept constant across treatments to allow comparison. Signal intensity, indicative of ROS accumulation, was quantified in the root apex and elongation zone using ImageJ software. Three independent biological replicates were carried out for each treatment, analyzing a minimum of 50 seedlings per replicate.

### 4.6. Statistical Analysis

Three independent replicates were conducted for each experiment, each including at least 50 seedlings, with results expressed as the mean value (±standard error). Statistical analyses were performed by first testing for homogeneity of variances (Levene’s Median Test) and then analyzing the data using one-way ANOVA with Tukey’s post hoc test (*p* ≤ 0.05). Letters on the graphs indicate significant differences.

## 5. Conclusions

This study reveals that non-CG DNA methylation plays a crucial role in modulating root responses to cadmium stress in *Arabidopsis thaliana*. The *ddc* mutant, defective in *DRM1/2* and *CMT3*, displayed enhanced tolerance through the preservation of auxin signaling, reduced ROS accumulation, and maintenance of quiescent center cell niche, probably due to a preserved auxin maxima and a fine-tuned ROS biosynthesis under cadmium stress. These findings support the idea that DNA methylation shapes the hormonal and oxidative landscape of the root meristem, thereby promoting developmental resilience under heavy metal exposure.

## Data Availability

The original contributions presented in this study are included in the article. Further inquiries can be directed to the corresponding authors.

## Abbreviations

The following abbreviations are used in this manuscript:

ABA	Abscisic acid
APX	Ascorbate peroxidase
CAT	Catalase
Cd^2+^	Cadmium
CdCl_2_	Cadmium chloride
CMT3	CHROMOMETHYLASE 3
Ctrl	Control
DAPI	4′,6-Diamidino-2-phenylindole
*ddc*	*drm1 drm2 cmt3* triple mutant
DRM1/DRM2	DOMAINS REARRANGED METHYLTRANSFERASE 1/2
GFP	Green fluorescent protein
GPX	Glutathione peroxidase
H_2_DCFDA	2′,7′-Dichlorodihydrofluorescein diacetate
H_2_O_2_	Hydrogen peroxide
IAA	Indole-3-acetic acid
IOD	Integrated optical density
JA	Jasmonic acid
MAPKs	Mitogen-activated protein kinases
MET1	METHYLTRANSFERASE 1
NO	Nitric oxide
O_2_•^−^	Superoxide radical
PIN	PIN-FORMED proteins
*PLT*	*PLETHORA*
QC	Quiescent center
RAM	Root apical meristem
RBOHD	Respiratory burst oxidase homolog D
ROS	Reactive oxygen species
SA	Salicylic acid
*SCR*	*SCARECROW*
WT	Wild type
*WOX5*	*WUSCHEL-RELATED HOMEOBOX 5*
*WUS*	*WUSCHEL*

## References

[1] Khan Z., Elahi A., Bukhari D.A., Rehman A. (2022). Cadmium sources, toxicity, resistance and removal by microorganisms-A potential strategy for cadmium eradication. J. Saudi Chem. Soc..

[2] Sharma R.K., Archana G. (2016). Cadmium minimization in food crops by cadmium resistant plant growth promoting rhizobacteria. Appl. Soil Ecol..

[3] Gill S.S., Hasanuzzaman M., Nahar K., Macovei A., Tuteja N. (2013). Importance of nitric oxide in cadmium stress tolerance in crop plants. Plant Physiol. Biochem..

[4] Bolan N., Mahimairaja S., Kunhikrishnan A., Naidu R. (2013). Sorption–bioavailability nexus of arsenic and cadmium in variable-charge soils. J. Hazard. Mater..

[5] Li P., Zhao C., Zhang Y., Wang X., Wang X., Wang J., Wang F., Bi Y. (2016). Calcium alleviates cadmium-induced inhibition on root growth by maintaining auxin homeostasis in *Arabidopsis* seedlings. Protoplasma.

[6] Bruno L., Pacenza M., Forgione I., Lamerton L.R., Greco M., Chiappetta A., Bitonti M.B. (2017). In *Arabidopsis thaliana* cadmium impact on the growth of primary root by altering *SCR* expression and auxin-cytokinin cross-talk. Front. Plant Sci..

[7] Bruno L., Talarico E., Madeo M.L., Muto A., Minervino M., Araniti F., Bitonti M.B., Chiappetta A. (2021). Cadmium affects cell niches maintenance in *Arabidopsis thaliana* post-embryonic shoot and root apical meristem by altering the expression of WUS/WOX homolog genes and cytokinin accumulation. Plant Physiol. Biochem..

[8] Lee J.Y., Benfey P.N., Roberts K. (2007). Root apical meristems. Handbook of Plant Science, 2 Volume Set.

[9] López-González D., Bruno L., Díaz-Tielas C., Lupini A., Aci M.M., Talarico E., Madeo M.L., Muto A., Sánchez-Moreiras A.M., Araniti F. (2023). Short-Term Effects of Trans-Cinnamic Acid on the Metabolism of *Zea mays* L. Roots. Plants.

[10] Lukačová Z., Švubová R., Kohanová J., Lux A. (2013). Silicon mitigates the Cd toxicity in maize in relation to cadmium translocation, cell distribution, antioxidant enzymes stimulation and enhanced endodermal apoplasmic barrier development. Plant Growth Regul..

[11] Choppala G., Saifullah B.N., Bibi S., Iqbal M., Rengel Z., Kunhikrishnan A., Ashwath N., Ok Y.S. (2014). Cellular mechanisms in higher plants governing tolerance to cadmium toxicity. Crit. Rev. Plant Sci..

[12] Shahid M., Pourrut B., Dumat C., Nadeem M., Aslam M., Pinelli E. (2014). Heavy-metal-induced reactive oxygen species: Phytotoxicity and physicochemical changes in plants. Rev. Environ. Contam. Toxicol..

[13] Benavides M.P., Gallego S.M., Tomaro M.L. (2005). Cadmium toxicity in plants. Braz. J. Plant Physiol..

[14] Araniti F., Talarico E., Madeo M.L., Greco E., Minervino M., Álvarez-Rodríguez S., Muto A., Ferrari M., Chiappetta A., Bruno L. (2023). Short-term exposition to acute cadmium toxicity induces the loss of root gravitropic stimuli perception through PIN2-mediated auxin redistribution in *Arabidopsis thaliana* (L.) *Heynh*. Plant Sci..

[15] Bruno L., Talarico E., Cabeiras-Freijanes L., Madeo M.L., Muto A., Minervino M., Lucini L., Miras-Moreno B., Sofo A., Araniti F. (2021). Coumarin Interferes with Polar Auxin Transport Altering Microtubule Cortical Array Organization in *Arabidopsis thaliana* (L.) *Heynh* Root Apical Meristem. Int. J. Mol. Sci..

[16] Haghpanah M., Namdari A., Kaleji M.K., Nikbakht-dehkordi A., Arzani A., Araniti F. (2025). Interplay Between ROS and Hormones in Plant Defense Against Pathogens. Plants.

[17] Novikova O. (2009). Chromodomains and LTR retrotransposons in plants. Commun. Integr. Biol..

[18] Talarico E., Zambelli A., Araniti F., Greco E., Chiappetta A., Bruno L. (2024). Unravelling the epigenetic code: DNA methylation in plants and its role in stress response. Epigenomes.

[19] Garro M., Greco E., Vannay G.J., Leonova A., Bruno L., Capella M. (2025). Non-CG DNA methylation represses SDC expression to modulate hypocotyl elongation during thermormorphogenesis in *Arabidopsis*. J. Exp. Bot..

[20] Forgione I., Wołoszyńska M., Pacenza M., Chiappetta A., Greco M., Araniti F., Abenavoli M.R., Van Lijsebettens M., Bitonti M.B., Bruno L. (2019). Hypomethylated *drm1 drm2 cmt3* mutant phenotype of *Arabidopsis thaliana* is related to auxin pathway impairment. Plant Sci..

[21] Forgione I., Muto A., Woloszynska M., Chiappetta A., Ferrari M., Van Lijsebettens M., Bitonti M.B., Bruno L. (2022). Epigenetic mechanisms affect the curled leaf phenotype in the hypomethylated *ddc* mutant of *Arabidopsis thaliana*. Plant Sci..

[22] Pacenza M., Muto A., Chiappetta A., Mariotti L., Talarico E., Picciarelli P., Picardi E., Bruno L., Bitonti M.B. (2021). In *Arabidopsis thaliana* Cd differentially impacts on hormone genetic pathways in the methylation defective *ddc* mutant compared to wild type. Sci. Rep..

[23] Wang Y., Sun X., Peng J., Li F., Ali F., Wang Z. (2025). Regulation of seed germination: ROS, epigenetic, and hormonal aspects. J. Adv. Res..

[24] Satoh-Nagasawa N., Mori M., Nakazawa N., Kawamoto T., Nagato Y., Sakurai K., Takahashi H., Watanabe A., Akagi H. (2012). Mutations in rice (*Oryza sativa*) heavy metal ATPase 2 (*OsHMA2*) restrict the translocation of zinc and cadmium. Plant Cell Physiol..

[25] Ronzan M., Piacentini D., Fattorini L., Della Rovere F., Eiche E., Riemann M., Altamura M.M., Falasca G. (2018). Cadmium and arsenic affect root development in *Oryza sativa* L. negatively interacting with auxin. Environ. Exp. Bot..

[26] Lee Y., Lee W.S., Kim S.H. (2013). Hormonal regulation of stem cell maintenance in roots. J. Exp. Bot..

[27] Heyman J., Kumpf R.P., De Veylder L. (2014). A quiescent path to plant longevity. Trends Cell Biol..

[28] Fattorini L., Ronzan M., Piacentini D., Della Rovere F., De Virgilio C., Sofo A., Altamura M.M., Falasca G. (2017). Cadmium and arsenic affect quiescent centre formation and maintenance in *Arabidopsis thaliana* post-embryonic roots disrupting auxin biosynthesis and transport. Environ. Exp. Bot..

[29] Roychoudhry S., Kepinski S. (2022). Auxin in root development. Cold Spring Harb. Perspect. Biol..

[30] Ding Z.J., Friml J. (2010). Auxin regulates distal stem cell differentiation in Arabidopsis roots. Proc. Natl. Acad. Sci. USA.

[31] Aida M., Beis D., Heidstra R., Willemsen V., Blilou I., Galinha C., Nussaume L., Noh Y.S., Amasino R., Scheres B. (2004). The *PLETHORA* genes mediate patterning of the *Arabidopsis* root stem cell niche. Cell.

[32] Eljebbawi A., Dolata A., Strotmann V.I., Stahl Y. (2024). Stem cell quiescence and dormancy in plant meristems. J. Exp. Bot..

[33] Sabatini S., Heidstra R., Wildwater M., Scheres B. (2003). *SCARECROW* is involved in positioning the stem cell niche in the *Arabidopsis* root meristem. Genes Dev..

[34] Zhai H., Zhang X., You Y., Lin L., Zhou W., Li C. (2020). SEUSS integrates transcriptional and epigenetic control of root stem cell organizer specification. EMBO J..

[35] Huang H., Ullah F., Zhou D.X., Yi M., Zhao Y. (2019). Mechanisms of ROS regulation of plant development and stress responses. Front. Plant Sci..

[36] Heyno E., Klose C., Krieger-Liszkay A. (2008). Origin of cadmium-induced reactive oxygen species production: Mitochondrial electron transfer versus plasma membrane NADPH oxidase. New Phytol..

[37] Cho U.H., Sohn J.Y. (2004). Cadmium-induced changes in antioxidative systems, hydrogen peroxide content, and lipid peroxidation in *Arabidopsis thaliana*. J. Plant Biol..

[38] Zhang T., Xiao J., Zhao Y., Zhang Y., Jie Y., Shen D., Yue C., Huang J., Hua Y., Zhou T. (2021). Comparative physiological and transcriptomic analyses reveal ascorbate and glutathione coregulation of cadmium toxicity resistance in wheat genotypes. BMC Plant Biol..

[39] Gutiérrez-Martínez P.B., Torres-Morán M.I., Romero-Puertas M.C., Casas-Solís J., Zarazúa-Villaseñor P., Sandoval-Pinto E., Ramírez-Hernández B.C. (2020). Assessment of antioxidant enzymes in leaves and roots of *Phaseolus vulgaris* plants under cadmium stress. Biotecnia.

[40] Liu Y.T., Chen Z.S., Hong C.Y. (2011). Cadmium-induced physiological response and antioxidant enzyme changes in the novel cadmium accumulator, *Tagetes patula*. J. Hazard. Mater..

[41] Pasternak T., Palme K., Pérez-Pérez J.M. (2023). Role of reactive oxygen species in the modulation of auxin flux and root development in *Arabidopsis thaliana*. Plant J..

[42] Bruno L., Araniti F., Talarico E., Greco E., Muto A., Pacenza M., Chiappetta A., Bitonti M.B. (2025). Transcriptomic and metabolomics analysis of the main stress-related pathways in the DNA methylation-defective *ddc* mutant of *Arabidopsis thaliana* exposed to Cd. Plant Biosyst..

[43] Truernit E., Bauby H., Dubreucq B., Grandjean O., Runions J., Barthélémy J., Palauqui J.C. (2008). High-resolution whole-mount imaging of three-dimensional tissue organization and gene expression enables the study of phloem development and structure in *Arabidopsis*. Plant Cell.

